# Chiniodine in Intermittent Fever

**Published:** 1851-05

**Authors:** Lewis Slusser

**Affiliations:** Canal Fulton, Ohio


### ARTICLE II.

Chiniodine in Intermittent Fever.—By Lewis Slusser, M. D., of Canal Fulton, Ohio.

Interesting as may be the history of rare and anomalous cases, they afford less of that which is of practical utility, than contributions upon diseases of more common occurrence.

Actuated by this belief, I am induced to make public my experience in the use of Chiniodine in the treatment of intermittent fever. The present high price of the alkaloid quinine, and the probability of its being maintained, render it an object of no small pecuniary consideration to country practitioners, who are compelled to dispense their own medicines, to obtain some reliable substitute less expensive.

Malarious fever is the endemic of this locality, and among its subjects, are a full quota of cases denominated charitable. The same remark will apply to many other sections of our country. For the last several years, impelled by motives of economy, as well as by a desire of professional improvement, I have *experimented* not a little with reputed anti-periodics—officinal and non-officinal. It would be an endless task, were I to attempt enumerating them. It may be proper, however, here to mention, inasmuch as chloroform is at present going the rounds as a succedaneum, that soon after its introduction into practice, I was led, from a knowledge of its power over the nervous system, to give it a trial; which I did in repeated cases, in different forms, and in varied combination; but found it entirely inefficacious—unworthy of the least confidence.

In the treatment of simple, uncomplicated intermittent, of a tertian or quartan type, I have as yet found no substitute equal to chiniodine. In quotidian cases, I have not succeeded so well; from the circumstance, as I believe, that the short intermissions will not allow sufficient time to bring the system under its influence. Where I could induce the patient to continue its exhibition through several intermissions, the disease invariably succumbed.

By medical statistics, or numerical analysis as it is termed by some writers, we are enabled to determine satisfactorily the value of a certain remedy in the cure of a specific disease. Success in a few isolated cases, is not sufficient to establish the merits or demerits of any article. A non-observance of this fact has often tended to bring reproach upon the writer, and consign his vaunted remedy to unmerited oblivion.

I have kept an accurate record of forty-two cases of intermittent, treated exclusively with chiniodine. These were of persons residing near at hand, coming almost daily under my observation, and the results of which I could note with confidence. Itinerant cases, I did not record. Of these forty-two cases, twenty-six were quartans; the balance, sixteen, were tertians. In all, when given in the mode and manner I shall hereafter direct, it was checked, without the recurrence of another paroxysm. Of the quartans, two relapsed on the eighth, and three upon the twenty-eighth day. Of the tertians, one relapsed upon the ninth, and two upon the twenty-first day. All of these were again checked by the same mode of treatment, and with the residue, have thus far escaped a relapse. According to this table, the proportion of relapse cases, is less than one-fifth of the whole number; a result which I think will compare favorably with the quinine treatment.

Upon a principle in therapeutics, that a combination of several articles of like properties will increase the aggregate effects, I have for the last year been prescribing chinoidine in accordance with the following formula:—

℞ Chiniodine ℥j.

Tart. Acid,

Cinch. Rub.,

Cascaril. pulv.,

Val. Rad. pulv., *aa.* ʒij.

Ex. Quas.,

Ex. Gentianæ, *aa.* ℥ss.

Mucil. Acaciæ, q.s.

M. Divide into 480 pills.

Of these, I usually prescribe to an adult, sixteen, two to be taken every three hours, commencing immediately after the subsidence of the last paroxysm, and continued during the day time until all are taken. To guard against relapse, I order six on the twelfth and thirteenth day after, counting from the last paroxysm; and the same quantity repeated on the twenty-sixth and twenty-seventh day.

The preparation of chinoidine that I have employed, was that manufactured by Powers & Weightman, Philadelphia.

The combination with Comp. Ex. Colocynth recommended by them in a circular accompanying the article, I have found objectionable, as being apt to disturb the bowels. I have used it successfully in conjunction with piperine, but the combination given above I have found from experience to be the more efficacious in meeting the indications.

In conclusion, I would remark, that I consider chinoidine entitled to more consideration than it has yet received from the profession, as a successful and comparatively cheap remedy in the treatment of intermittent fever.—*Med. Exam.*

